# Polymorphisms in LPL, CETP, and HL protect HIV-infected patients from atherogenic dyslipidemia in an allele-dose-dependent manner

**DOI:** 10.7448/IAS.17.4.19557

**Published:** 2014-11-02

**Authors:** Patricia Echeverría, Montse Guardiola, Marta González, Joan Carles Vallvé, Jordi Puig, Anna Bonjoch, Bonaventura Clotet, Josep Ribalta, Eugenia Negredo

**Affiliations:** 1 Fundació Lluita contra la Sida, Hospital Universitari Germans Trias i Pujol, Badalona Universitat Autònoma de Barcelona, HIV, Barcelona, Spain; 2 Institut d'Investigació Sanitària Pere Virgili, Universitat Rovira i Virgili, Unitat de Recerca en Lípids i Arteriosclerosi, Barcelona, Spain; 3 Fundació Lluita contra la Sida, Hospital Universitari Germans Trias i Pujol, Badalona, HIV, Barcelona, Spain

## Abstract

**Introduction:**

HIV-infected patients treated with Highly Active Antiretroviral Therapy (HAART) may be predisposed to hypertriglyceridemia, which gives rise to a highly atherogenic lipid profile known as atherogenic dyslipidemia (AD). We propose that genetic variability leaves some HIV-infected patients more predisposed to AD than others [1, 2].

**Methods:**

This was a cross-sectional, observational study conducted in 468 antiretroviral-treated HIV-infected patients attending at the outpatient clinic of a tertiary hospital over a 6-month period, who were classified as normolipidemic (n=173) or presenting with AD (triglycerides: 1.7 mmol/L and HDLc < 1.02 [men] or 1.28 mmol/L [women]) (n=148). Polymorphisms were identified in the APOA5, APOC3, LPL, CETP, HL, MTP, APOE, LRP5 and VLDLR genes.

**Results:**

Atherogenic dyslipidemia was detected in 31% of patients, most of whom were men (77%). This group was also older and had higher levels of remnant lipoprotein cholesterol (RLPc) than normolipidemic patients. The polymorphisms rs328 in LPL, rs708272 in CETP and rs1800588 in HL were 10–40% significantly more frequent in normolipidemic patients. At least 1 of these polymorphisms was detected in 90% of normolipidemic patients; in AD patients, the percentage decreased to 75% (p=0.003). This effect was dependent on both the allele and the dose of HAART and independent of the regimen administered. The protective combination showed a trend towards higher HDLc (1.13 [0.40] vs 1.24 [0.23] mmol/L), lower triglycerides (2.23 [2.34] vs 1.89 [1.24] mmol/L) and lower RLPc (16.41 [11.42] vs 12.99 [11.69] mmol/L).

**Conclusions:**

Polymorphisms in LPL, CETP and HL protect HIV-infected patients from developing AD in a dose-dependent manner [3].
